# Volunteering, Health, and Well-being of Children and Adolescents in the United States

**DOI:** 10.1001/jamanetworkopen.2023.15980

**Published:** 2023-05-30

**Authors:** Kevin Lanza, Ethan T. Hunt, Dale S. Mantey, Onyinye Omega-Njemnobi, Benjamin Cristol, Steven H. Kelder

**Affiliations:** 1 University of Texas Health Science Center at Houston, School of Public Health in Austin, Michael & Susan Dell Center for Healthy Living, Austin

## Abstract

This cross-sectional study examines whether there is an association between volunteering and well-being among children and adolescents across the United States.

## Introduction

The positive effects of volunteering (ie, undertaking unpaid work to benefit others) on the health and well-being of adult volunteers are well-established.^1,2^ However, the effect of volunteering on youths is largely unknown, with 2 studies of limited samples of adolescent volunteers finding volunteering to reduce cardiovascular risk factors^3^ and to positively associate with school engagement.^4^ Herein, we assessed the association between volunteering and the health and well-being of children and adolescents across the United States.

## Methods

This cross-sectional study’s data originated from the publicly available 2019 to 2020 National Survey of Children’s Health.^5^ Data were adjusted and weighted to reflect the demographic composition of youths in each state. We used parent-reported survey data for children aged 6 to 11 years (n = 22 126) and adolescents aged 12 to 17 years (n = 29 769). Institutional review board approval and informed consent were not required because we did not merge and/or enhance the data set such that individuals might be identified. This study followed the STROBE reporting guideline.

Parents answered whether, during the past 12 months, their child or adolescent participated in community service or volunteer work at school, church, or in the community. The study had 5 parent-reported, dichotomous outcomes: (1) excellent and/or very good health, (2) flourishing, (3) anxiety, (4) depression, and (5) behavioral problems (eMethods in Supplement 1). We specified logit regression (n = 15 models) in Stata version 16 (StataCorp) to test the association between volunteering and the 5 outcomes for children, adolescents, and the total sample. Significance was set at a 2-sided *P* < .05. Statistical analysis was performed from March to April 2022. Models were adjusted for sex, race and ethnicity, household income, parental religiosity, and urbanicity (eMethods in Supplement 1).^6^

## Results

Among the 51 895 youths included in the sample, 22 126 were children aged 6 to 11 years, 29 769 were adolescents aged 12 to 17 years, 26 863 (52%) were male, 3621 (7%) were Black, 6618 (13%) were Hispanic, and 35 021 (67%) were White; most youths in the study were above poverty level (88%), metropolitan (83%), in excellent or very good health (64%), flourishing (64%), and without behavioral problems (90%) (Table 1). More adolescents than children reported anxiety (19% vs 11%), depression (11% vs 3%), and volunteering (54% vs 34%). In modeling (Table 2), volunteering was associated with higher odds of parent-reported excellent or very good health in children (adjusted odds ratio [aOR], 1.25; 95% CI, 1.07-1.47; *P* = .006) and adolescents (aOR, 1.42; 95% CI, 1.24-1.63; *P* < .001). Volunteering was also associated with higher odds of flourishing in children (aOR, 1.35; 95% CI, 1.15-1.58; *P* < .001) and adolescents (aOR, 1.97; 95% CI, 1.72-2.26; *P* < .001). There was no association between volunteering and anxiety for children (aOR, 1.02; 95% CI, 0.81-1.28; *P* = .88), while volunteering was associated with lower odds of anxiety in adolescents (aOR, 0.74; 95% CI, 0.61-0.88; *P* = .001). There was no association between volunteering and depression for children (aOR, 0.74; 95% CI, 0.48-1.15; *P* = .18) and adolescents (aOR,0.78; 95% CI, 0.60-1.01; *P* = .06). Volunteering was associated with lower odds of behavioral problems in children (aOR, 0.64; 95% CI, 0.51-0.79; *P* < .001) and adolescents (aOR, 0.67; 95% CI, 0.54-0.83; *P* < .001).

**Table 1.** Summary Statistics for Children, Adolescents, and Total Sample

Model variable	No. (%)		
	Children (aged 6-11 y) (n = 22 126)	Adolescents (aged 12-17 y) (n = 29 769)	Total sample (aged 6-17 y) (N = 51 895)
Sex			
Female	10 686 (48)	14 346 (48)	25 032 (48)
Male	11 440 (52)	15 423 (52)	26 863 (52)
Race and ethnicity			
Asian	1162 (5)	1591 (5)	2753 (5)
Black	1579 (7)	2042 (7)	3621 (7)
Hispanic	2868 (13)	3750 (13)	6618 (13)
White	14 682 (67)	20 339 (68)	35 021 (67)
Other^a^	1835 (8)	2047 (7)	3882 (7)
Household income			
Below federal poverty level	2762 (12)	3390 (11)	6152 (12)
Parental religiosity			
Support from place of worship/religious leader	4857 (23)	7006 (24)	11 863 (23)
Urbanicity			
Metropolitan statistical area	13 036 (84)	17 364 (83)	30 400 (83)
Health			
Excellent/very good health	14 829 (67)	18 552 (62)	33 381 (64)
Flourishing	13 759 (63)	19 006 (64)	32 765 (64)
Anxiety	2372 (11)	5476 (19)	7848 (15)
Depression	573 (3)	3200 (11)	3773 (7)
Behavioral problems	2457 (11)	2865 (10)	5322 (10)
Volunteering			
Participation in community service/volunteer work	7453 (34)	15 739 (54)	23 192 (46)

^a^ Other race and ethnicity categories included American Indian and Alaska Native, Native Hawaiian and Pacific Islander, and multiracial.

**Table 2.** Adjusted Regression Models Examining Odds of Excellent or Very Good Health, Flourishing, Anxiety, Depression, and Behavioral Problems Given History of Volunteering^a^

Model outcome	Children (ages 6-11 y)		Adolescents (ages 12-17 y)		Total sample (ages 6-17 y)	
	aOR (95% CI)	*P* value	aOR (95% CI)	*P* value	aOR (95% CI)	*P* value
Excellent or very good health	1.25 (1.07-1.47)	.006	1.42 (1.24-1.63)	<.001	1.34 (1.21-1.49)	<.001
Flourishing	1.35 (1.15-1.58)	<.001	1.97 (1.72-2.26)	<.001	1.66 (1.50-1.84)	<.001
Anxiety	1.02 (0.81-1.28)	.88	0.74 (0.61-0.88)	.001	0.82 (0.71-0.95)	.01
Depression	0.74 (0.48-1.15)	.18	0.78 (0.60-1.01)	.06	0.78 (0.62-0.98)	.03
Behavioral problems	0.64 (0.51-0.79)	<.001	0.67 (0.54-0.83)	<.001	0.65 (0.56-0.76)	<.001

Abbreviation: aOR, adjusted odds ratio.

^a^ Independent variable of interest: participation in community service or volunteer work during the past 12 months. All models adjusted for sex, race and ethnicity, household income, parental religiosity, urbanicity (not shown). All variables were dichotomous.

## Discussion

Using survey data from across the United States, we found that volunteering was associated with higher odds of excellent or very good health and flourishing in children and adolescents, and with lower odds of anxiety in adolescents and behavioral problems in children and adolescents. Study limitations included lack of a diverse sample, potential response bias from parent-reported data, and the cross-sectional design not permitting determination of causality.

This study’s findings are encouraging of further investigation to assess causality, which if revealed, may provide opportunity for prescribing volunteering as a public health intervention. Furthermore, with volunteering in adolescence having been found to be associated with decreases in risky health behaviors and depressive symptoms in adulthood,^6^ youths who help others may be helping themselves now and later.

## Notes

Supplement 1. **eMethods.** Study Variables Developed from 2019-2020 National Survey of Children’s Health (NSCH)

Supplement 2.
Data Sharing Statement
