# Supplementary material for: Volunteering, Health, and Well-being of Children and Adolescents in the United States
Source: JAMA Netw Open. 2023 May 30;6(5):e2315980. doi: 10.1001/jamanetworkopen.2023.15980 (PMC10230312; doi:10.1001/jamanetworkopen.2023.15980)
Supplement: Supplement 1. — eMethods. Study Variables Developed from 2019-2020 National Survey of Children’s Health (NSCH) [file jamanetwopen-e2315980-s001.pdf]

Supplemental Online Content

Lanza K, Hunt ET, Mantey DS, Omega-Njemnobi O, Cristol B, Kelder SH. Volunteering, health, and well-being of children and adolescents in the United States. *JAMA Netw Open.* 2023;6(5):e2315980. doi:10.1001/jamanetworkopen.2023.15980

eMethods. Study Variables Developed from 2019-2020 National Survey of Children's Health (NSCH)

This supplemental material has been provided by the authors to give readers additional information about their work.

© 2023 Lanza K et al. *JAMA Network Open.*

eMethods. Study Variables Developed from 2019-2020 National Survey of Children's Health (NSCH)

parent-reported survey data for children and adolescents

Volunteering: 0 = no participation in community service or volunteer work in past 12 months, 1 = participation in community service or volunteer work in past 12 months. Developed from NSCH variable volunteer_1920.

Excellent/very good health: 0 = overall health status good, fair, or poor; 1 = overall health status excellent or very good. Developed from NSCH variable k2q01.

Flourishing: 0 = does not meet all three flourishing items, 1 = meets all three flourishing items (flourishing items: children who 1) show interest and curiosity in learning new things, 2) work to finish the tasks they start, 3) stay calm and in control when faced with challenges). Developed from NSCH variable flrish6to17_1920.

Anxiety: 0 = no anxiety, 1 = anxiety. Developed from NSCH variable anxiety_1920.

Depression: 0 = no depression, 1 = depression. Developed from NSCH variable depress_1920.

Behavioral problems: 0 = no behavior or conduct problems, 1 = behavior or conduct problems. Developed from NSCH variable behavior_1920.

Female: 0 = male, 1 = female. Developed from NSCH variable sex_1920.

Asian: 0 = not Asian, 1 = Asian (referent = white). Developed from NSCH variable raceASIA_1920.

Black: 0 = not Black, 1 = Black (referent = white). Developed from NSCH variable raceASIA_1920.

Hispanic: 0 = not Hispanic, 1 = Hispanic (referent = white). Developed from NSCH variable raceASIA_1920.

Other: 0 = not American Indian, Alaska Native, Native Hawaiian, other Pacific Islander, or Multi-Racial, 1 = American Indian, Alaska Native, Native Hawaiian, other Pacific Islander, or Multi-Racial, (referent = white). Developed from NSCH variable raceASIA_1920.

Household income: 1 = 0-99% federal poverty level, 2 = 100%-199% federal poverty level, 3 = 200%-399% federal poverty level, 4 = 400% federal poverty level or greater. Developed from NSCH variable povlev4_1920.

Parental religiosity: 0 = parent did not receive day-to-day emotional support from a place of worship/religious leader, 1 = parent did receive day-to-day emotional support from a place of worship/religious leader. Developed from NSCH variable EmSWorship_1920.

Urbanicity: 0 = not metropolitan statistical area, 1 = metropolitan statistical area. Developed from NSCH variable metro_yn.

© 2023 Lanza K et al. *JAMA Network Open.*
