# Supplementary material for: Volunteering, Health, and Well-being of Children and Adolescents in the United States
Source: JAMA Netw Open. 2023 May 30;6(5):e2315980. doi: 10.1001/jamanetworkopen.2023.15980 (PMC10230312; doi:10.1001/jamanetworkopen.2023.15980)
Supplement: Supplement 2. — Data Sharing Statement [file jamanetwopen-e2315980-s002.pdf]

Data Sharing Statement

Lanza. Volunteering, Health, and Well-being of Children and Adolescents in the United States. *JAMA Netw Open*. Published May 30, 2023. doi:10.1001/jamanetworkopen.2023.15980

Data
Data available: Yes
Data types: Deidentified participant data, Data dictionary
How to access data: Data are available by request from Kevin Lanza (Kevin.L.Lanza@uth.tmc.edu).
When available: With publication

Supporting Documents
Document types: Statistical/analytic code
How to access documents: Analytic code is available by request from Kevin Lanza (Kevin.L.Lanza@uth.tmc.edu).
When available: With publication

Additional Information
Who can access the data: Data will be made available for anyone requesting the data.
Types of analyses: Data will be made available for any analytical purpose.
Mechanisms of data availability: The data will be made available without investigator support.
